# Epicardial adipose tissue volume and arterial stiffness in people living with diabetes: the METAB-CV-PWV study

**DOI:** 10.1186/s12933-025-02933-z

**Published:** 2025-10-15

**Authors:** Omar Nouhou Koutcha, Sopio Tatulashvili, Nanthara Sritharan, Pierre Boutouyrie, Imen Rezgani, Marouane Boubaya, Mohamed Lamine Mariko, Lucie Allard, Meriem Sal, Coralie Bloch-Queyrat, Mohamed Zerguine, Pierre-Yves Brillet, Zouhour El Fekih, Rosa-Maria Bruno, Hélène Bihan, Emmanuel Cosson

**Affiliations:** 1 Department of Endocrinology-Diabetology-Nutrition, CRNH-IdF, CSO IDF Nord, Avicenne Hospital, AP-HP, Université Paris 13, Sorbonne Paris Cité, 125 Rue de Stalingrad, 93000 Bobigny, France; 2 Clinical Research Unit, Avicenne Hospital, AP-HP, Bobigny, France; 3 Department of Pharmacology-Toxicology, Georges Pompidou European Hospital, AP-HP, Paris, France; 4 Department of Radiology, Avicenne Hospital, AP-HP, Bobigny, France; 5 Hypertension Unit, Georges Pompidou European Hospital, AP-HP, Paris, France; 6 Laboratoire Educations et Pratiques de Santé UR 3412, UFR Santé, Médecine, Biologie Humaine, Université Paris Sorbonne Paris Nord, 74, rue Marcel Cachin, 93017 Bobigny Cedex, France; 7 Center of Research in Epidemiology and StatisticS (CRESS), Nutritional Epidemiology Research Team (EREN), INSERM, INRAE, CNAM, Université Sorbonne Paris Nord and Université Paris Cité, 93017 Bobigny, France

**Keywords:** Arterial stiffness, Epicardial adipose tissue, Cardiovascular risk, Diabetes, Pulse wave velocity

## Abstract

**Introduction:**

Epicardial adipose tissue (EAT) and arterial stiffness are determinants of excess risk of cardiovascular disease in persons with diabetes. This study aimed to evaluate the relationship between both of these conditions in a cohort of patients with diabetes.

**Materials and methods:**

A part retrospective, part prospective non-interventional cohort study of people living with diabetes who had (i) a computed tomography scan to measure both their coronary artery calcium score and EAT volume (proprietary prototype, GE HealthCare), and (ii) a finger-to-toe pulse wave velocity (PWV) measurement to assess arterial stiffness. The study’s ClinicalTrials.gov identifier is NCT05681533.

**Results:**

A total of 345 participants (198 men, mean age (± standard deviation (SD)) 55.6 ± 12.6 years) were included; 73.6% had type 2 diabetes and 41.6% had obesity. Median duration of diabetes was 12 [interquartile range (IQR) 6–20] years. The median PWV was 8.0 [IQR 7.0–10.0] m/sec and median EAT volume was 84.9 [IQR 61.8–114.3] cm^3^. A positive correlation was observed between EAT volume and PWV (r = 0.37 [95% confidence interval (95%CI) 0.27–0.45], *p* < 0.001). EAT volume was associated with PWV tertile: specifically, participants in the first (≤ 7.0 m/sec), second, and third (> 9.0 m/sec) tertiles had, respectively, EAT volumes of 76.3 [IQR 50.3–100.6] cm^3^, 82.5 [IQR 64.4–107.3] cm^3^, and 100.2 [IQR 77.3–134.6] cm^3^ (*p* < 0.001 for all three). After adjustment for age, mean blood pressure, body mass index and diabetes type, each 10 cm^3^ increase in EAT volume was associated with a 14% increase in the probability of belonging to the third PWV tertile (odds ratio 1.14 [95%CI 1.06 – 1.21]; *p* < 0.001).

**Conclusion:**

EAT volume was associated with arterial stiffness in people living with diabetes. This association suggests that systemic inflammatory and metabolic mechanisms, through EAT and/or other associated ectopic adipose tissues, may contribute to an increased risk of cardiovascular disease.

**Supplementary Information:**

The online version contains supplementary material available at 10.1186/s12933-025-02933-z.

## Introduction

Despite great advances in related healthcare over recent years, diabetes is still associated with a higher risk of cardiovascular morbidity and mortality [1–3]. In 2021, more than six million people worldwide died from diabetes-related complications, including cardiovascular disease and heart failure [3]. In order to reduce cardiovascular morbidity and mortality associated with diabetes, their pathophysiology needs to be better understood and new risk markers identified. Two potential markers are epicardial adipose tissue (EAT) [4–8] and arterial stiffness [9–13].

EAT is located between the myocardium and the visceral layer of the pericardium; it is considered the visceral adipose tissue of the cardiac system [6, 7, 14]. It secretes inflammatory factors and lipid metabolites, which may be determinants of accelerated atherosclerosis [6–8, 15]. A recent meta-analysis showed that people with diabetes —whether type 1 (T1D) or type 2 (T2D)—had higher EAT volumes than healthy controls, irrespective of the method used to quantify EAT volume [5]. We recently showed that EAT volume in persons with diabetes was associated with a higher coronary artery calcium (CAC) score [16] and a higher percentage of myocardial ischemia [17]. In addition, EAT thickness has been positively associated with the risk of incident cardiovascular events in persons with T2D [4].

The Expert Consensus on Arterial Stiffness defined pulse wave velocity (PWV) as the gold standard measurement for assessing arterial stiffness, as it is relatively ease to determine and is reliable [9–11]. Higher PWV is associated with higher rates of incident cardiovascular disease in the general population [10, 12, 13] and in persons with diabetes [18].

To date, relatively few studies have assessed the relationship between EAT volume (or thickness) and arterial stiffness for diseases in general [19–30]. Of these, only three comprised specifically persons with T2D [19, 29, 30]. In the former studies, two assessed aortic compliance [29, 30] and not PWV [19]. These studies had relatively small sample sizes and could adjust their results only for a few confounding factors, mixing patients with T2D and their controls. In this context, the present study aimed to evaluate the relationship between EAT volume and arterial stiffness by PWV measurement in a series of persons with type 2, type 1 and other types of diabetes large enough to adjust for potential confounders.

## Methods

### Inclusion

METAB-CV-PWV is a part retrospective, part prospective non-interventional cohort study designed to evaluate the relationship between EAT and arterial stiffness in persons with diabetes. We included non-pregnant inpatient and outpatient adults with diabetes from our Endocrinology-Diabetology-Nutrition department in Avicenne Hospital in Bobigny, France. All persons in the cohort underwent two routine departmental procedures: (i) a computed tomography (CT) scan to measure the CAC score, and (ii) a finger-to-toe PWV measure with pOpmetre® to measure arterial stiffness [16, 31]. Both these examinations are routinely performed to evaluate cardiovascular risk in our department [16, 31]. For the present study, the CT scans were also used to measure EAT volume [16, 17, 32]. Patients with peripheral artery disease were not included because it might influence PWV measurement [9–11].

The study was approved by the local ethics committee in September 2021 (CLEA-2021-204). We retrospectively included patients who had both the CT scan and PWV measurement between April 2021 and September 2021. Potential participants were sent an information sheet by post detailing the study, its objectives, and their rights; those who did not express opposition were included. We also prospectively included patients who had these two examinations between October 2021 and September 2022, and who also immediately indicated their non-opposition when they had their PWV measurement. We excluded patients who had a poor-quality PWV measurement and those whose EAT volume could not be determined from the CT scan (Supplementary Fig. 1, Flow chart). A PWV measurement is considered poor quality if atypical or unusable waveforms prevent a reliable transit time calculation. In practice, this corresponds to irregular or noisy waveforms caused by movement, arrhythmia or poor sensor contact, an absence of clear foot detection on one of the pulse signals, or significant asymmetry between the right and left signals.

The study was registered on ClinicalTrials.gov under the following identifier: NCT05681533. It adhered to the ethical principles outlined in the Declaration of Helsinki.

### Data collection

Data were extracted from patients’ medical records and collected anonymously in a secure health database general data such as the type of diabetes (T1D, T2D or other types as written in medical records), premature (i.e., < 55 years and < 65 years for men and women, respectively) coronary artery disease diagnosis in first degree relatives; medical history: hypertension and dyslipidemia were self-reported and/or inferred from prescriptions for antihypertensive and lipid-lowering agents, respectively. We collected information on retinopathy (detected by fundus photography or ophthalmoscopy), nephropathy (defined as renal failure (i.e., an estimated glomerular filtration rate [eGFR] < 60 ml/min) and/or micro or macroalbuminuria), neuropathy (defined as any sign or symptom of polyneuropathy), and macroangiopathy (history of acute coronary syndrome, stroke, peripheral artery occlusive disease, 50% or greater stenosis measured by ultrasound examination).

We collected information on glycated hemoglobin (HbA1c) (calculated using high performance liquid chromatography), total and HDL-cholesterol (calculated using a colorimetric assay on homogenous phase and cholesterol dosage by cholesterol oxidase), triglycerides (colorimetric assay), and LDL-cholesterol (calculated using the Friedewald formula). All these measurements were performed on plasma from fasting individuals using a Cobas 6000 analyzer (Roche diagnostics, Meylan, France). We also measured serum creatinine (using colorimetry, Kone Optima, Thermolab System, Paris La Défense, France) and estimated the glomerular filtration rate (using the chronic kidney disease-Epidemiology Collaboration equation). Moreover, we measured the urinary albumin excretion rate (using immunoturbidimetry, Cobas c501, Roche Diagnostics, Meylan, France), with levels between 30 and 299 mg/24 h defining microalbuminuria, and higher levels defining macroalbuminuria.

### Arterial stiffness

We used pOpmetre® to non-invasively measure arterial stiffness [33–35]. It is a certified Class IIa Medical Device (under European regulation). It has been validated against the reference method, the carotid–femoral PWV assessed by applanation tonometry, and shows a strong correlation and good reproducibility. Repeatability was good (coefficient of variation: 4.52%, mean difference: 0.02–0.50 m/sec), and obtained over a wide range of PWV (5–28 m/sec) [33]. This PWV measurement device employs two photo diode sensors, similar to pulse oximeters; one is positioned on the tip of a finger and the other on a toe. pOpmetre® measures the transit time of a pulse between the finger and toe; this is considered to be an approximation of the transit time of the aortic pulse [33, 34]. Pulse transit times are measured continuously for 20 s. The pulse wave transit time between the toe and finger (ft-TT, in milliseconds) is used to calculate the PWV (in meters per second: PWV = K x patient height/ft-TT), where K is a height-dependent constant [33, 34, 36]. The higher the PWV, the greater the arterial thickness.

### CT imaging

We calculated EAT volume and CAC score using ECG-gated cardiac CT without contrast injection. All CT scans were performed with General Electric (GE, HealthCare Digital, France) or Siemens (Healthineers, France) scanners. EAT volume was estimated a semi-automatic segmentation technique on every axial slice from the thoracic inlet to the beginning of the abdomen. We used the Volume Viewer software package (proprietary prototype, GE HealthCare). In the first step, an artificial intelligence-based algorithm segments the heart envelope. In the second step, a histogram-based method extracts two classes of pixels inside the envelope, corresponding to the EAT (values between -190 and -30 Hounsfield units) and the soft tissue [16, 17, 32]. EAT volume is expressed in cm^3^. Supplemental Fig. 2 shows an example measurement of EAT volume.

### Sample size calculation

Prior to METAB-CV-PWV, the only information in the literature regarding EAT volume and arterial stiffness as qualitative data came from a South Korean study which found EAT volumes of 125 cm^3^ for the third PWV tertile vs. 64 cm^3^ for the first/second tertiles in 111 patients with coronary artery disease, including 11% persons with diabetes [24]. We used the same methodology as in that study. In order to calculate the required sample size, we considered the results of the first 75 participants retrospectively included in our study: EAT volume was 105 cm^3^ (standard deviation (SD) = 42) in participants in the third PWV tertile vs. 88 cm^3^ (SD = 38) in participants in the other two tertiles. With a desired power of 95% and an alpha risk of 5%, we calculated that we would need 310 patients for the overall cohort study sample.

### Statistical analyses

Data were collected using the Research Electronic Data Capture platform (REDCap; Vanderbilt University, Nashville, TN). Categorical variables were described using numbers and percentages. For continuous variables, the median and interquartile range (IQR) or mean ± SD were used, depending on the distribution. To compare characteristics across PWV tertiles, we performed univariate tests, specifically Fisher’s exact test or the Chi-2 test for categorical variables, and the Kruskal–Wallis test for continuous variables. Spearman’s rank correlation was used to assess the relationship between PWV and EAT volume. A 95% confidence interval (95% CI) for the correlation coefficient was calculated using the bootstrap method. Analyses were performed for each of three diabetes type subgroups—T1D, T2D, all other types of diabetes—to explore potential inter-group differences.

Finally, to assess the association between a high arterial stiffness score (i.e., third PWV tertile) and EAT volume, we performed multivariable logistic regression models. The covariates included in Model 1 were age and central mean blood pressure—two established determinants of PWV [9–11]—as well as body mass index (BMI) and diabetes type—two major determinants of EAT volume in persons with diabetes (16,17). Results from Model 1 were stratified by obesity status (BMI ≥ 30 kg/m^2^ vs < 30 kg/m^2^). Model 2 added ethnicity to the covariates of Model 1, Model 3 further included lipid levels, Model 4 additionally included diabetes-related complications. In Model 5, central mean blood pressure was replaced with the presence of hypertension, while in Model 6, age was replaced with diabetes duration. We presented several models rather than a single fully adjusted model to avoid potential over-adjustment and because the sample size limited the number of covariates included in models. According to the rule of thumb of at least 10 events per variable, the maximum number of covariates was limited to 10 to ensure model stability. This sequential modeling approach also allows assessment of how the association of interest evolves with the addition of clinically relevant covariate blocks. Odds-ratios and their 95% CI were reported. Multiple imputation was performed using the Multiple Imputation by Chained Equation (MICE) method to account for missing data. Ten imputed datasets were generated, with twenty iterations per dataset. All analyses were performed based on imputed datasets by pooling estimates from each imputed dataset according to Rubin’s rules. All statistical analyses were carried out using R statistical software version 4.5.1 (R Foundation for Statistical Computing; URL https://www.R-project.org/). A *p*-value of < 0.05 was considered statistically significant.

## Results

### Inclusion and patient’s characteristics

The study flow chart is provided in supplementary Fig. 1 and Table 1 shows the study population’s characteristics. Of the 345 patients included, 198 (57.4%) were men, 254 had T2D, 66 had T1D, and 25 had other types of diabetes. The median diabetes duration was 12 years [IQR 6–20], and 52.2% of patients were taking insulin.

**Table 1:** Patients’ characteristics according to pulse wave velocity tertiles

	Available data	Total	PWV Tertile 1 ≤ 7	PWV Tertile 2 [7–9]	PWV Tertile 3 > 9	*p*
		n = 345	n = 136	n = 102	n = 107	
*Clinical characteristics*						
Mean age ± SD (years)	345	55.6 (12.6)	49.5 (13.5)	56.9 (10.5)	62.1 (9.2)	< 0.001
Male sex	345	198 (57.4%)	83 (61.0%)	55 (53.9%)	60 (56.1%)	0.52
Ethnicity	342					0.006
Caucasian		67 (19.6%)	28 (20.6%)	16 (16.0%)	23 (21.7%)	
African/Antilles		105 (30.7%)	44 (32.4%)	40 (40.0%)	21 (19.8%)	
North African		109 (31.9%)	38 (27.9%)	27 (27.0%)	44 (41.5%)	
Asiatic		49 (14.3%)	16 (11.8%)	16 (16.0%)	17 (16.0%)	
Other		12 (3.5%)	10 (7.4%)	1 (1.0%)	1 (0.9%)	
Obesity	327	136 (41.6%)	48 (38.7%)	39 (39.4%)	49 (47.1%)	0.31
Body mass index (kg/m^2^)	344	28.0 [25.0;32.0]	28.5 [24.0;33.0]	28.0 [25.0;32.0]	29.0 [25.0;32.0]	0.79
*Diabetes*						
Type	345					< 0.001
Type 2		254 (73.6%)	77 (56.6%)	86 (84.3%)	91 (85.0%)	
Type 1		66 (19.1%)	42 (30.9%)	10 (9.8%)	14 (13.1%)	
Other		25 (7.2%)	17 (12.5%)	6 (5.9%)	2 (1.9%)	
Time since diagnosis (years)	344	12.0 [6.0;20.0]	9.5 [2.0;18.2]	11.5 [8.0;19.0]	16.5 [10.0;22.0]	< 0.001
HbA1c (%)	290	8.4 [7.3;10.5]	8.9 [7.2;12.0]	8.4 [7.5;10.1]	8.1 [7.2;9.8]	0.046
*Diabetes-related treatment*						
Metformin	345	226 (65.5%)	74 (54.4%)	71 (69.6%)	81 (75.7%)	0.001
Sulfonylurea	345	120 (34.8%)	35 (25.7%)	40 (39.2%)	45 (42.1%)	0.02
Alpha-glucosidase inhibitor	345	8 (2.3%)	3 (2.2%)	3 (2.9%)	2 (1.9%)	> 0.9
Di-peptidyl-peptidase 4 inhibitor	345	76 (22.0%)	26 (19.1%)	28 (27.5%)	22 (20.6%)	0.28
Sodium-glucose cotransporter-2 inhibitor	345	19 (5.5%)	4 (2.9%)	7 (6.9%)	8 (7.5%)	0.24
Glucagon-like peptide 1 receptor agonists	345	85 (24.6%)	22 (16.2%)	28 (27.5%)	35 (32.7%)	0.01
Insulin	345	180 (52.2%)	70 (51.5%)	57 (55.9%)	53 (49.5%)	0.64
*Diabetes-related complications*						
Retinopathy	344	122 (35.5%)	39 (28.7%)	33 (32.4%)	50 (47.2%)	0.04
Estimated glomerular filtration rate ≥ 60 ml/min	341	307 (90.0%)	125 (94.7%)	89 (87.3%)	93 (86.9%)	0.07
Albuminuria	264					0.03
No		180 (68.2%)	74 (74.0%)	58 (75.3%)	48 (55.2%)	
Microalbuminuria		69 (26.1%)	23 (23.0%)	15 (19.5%)	31 (35.6%)	
Macroalbuminuria		15 (5.7%)	3 (3.0%)	4 (5.2%)	8 (9.2%)	
Nephropathy	344	83 (24.1%)	27 (19.9%)	22 (21.6%)	34 (32.1%)	0.16
Neuropathy	344	133 (38.7%)	42 (30.9%)	47 (46.5%)	44 (41.1%)	0.04
Macroangiopathy	342	37 (10.8%)	9 (6.7%)	9 (8.9%)	19 (17.9%)	0.02
*Additional cardiovascular risk factors*						
Family history of premature CAD	339	20 (5.9%)	6 (4.6%)	8 (7.8%)	6 (5.7%)	0.57
Hypertension*	344	174 (50.6%)	46 (33.8%)	55 (53.9%)	73 (68.9%)	< 0.001
Central systolic blood pressure, mmHg	305	136.0 [116.0;140.0]	127.0 [109.2;139.0]	137.0 [117.0;140.5]	139.0 [125.0;141.0]	< 0.001
Central diastolic blood pressure, mmHg	305	73.0 [72.0;77.0]	73.0 [72.0;78.8]	73.0 [72.0;76.5]	73.0 [72.0;76.0]	0.32
Mean central blood pressure, mmHg	305	94.7 [90.0;95.7]	94.0 [87.3;95.7]	94.7 [88.7;95.7]	95.0 [94.0;95.7]	0.03
Antihypertensive treatment						
Angiotensin-converting enzyme inhibitors	345	99 (28.7%)	30 (22.1%)	32 (31.4%)	37 (34.6%)	0.08
Angiotensin 2 receptor blockers	345	68 (19.7%)	15 (11.0%)	20 (19.6%)	33 (30.8%)	0.001
Beta blockers	345	50 (14.5%)	10 (7.4%)	15 (14.7%)	25 (23.4%)	0.002
Calcium channel inhibitors	345	84 (24.3%)	23 (16.9%)	29 (28.4%)	32 (29.9%)	0.03
Other	345	81 (23.5%)	17 (12.5%)	23 (22.5%)	41 (38.3%)	< 0.001
Dyslipidemia*	343	188 (54.8%)	52 (38.8%)	63 (61.8%)	73 (68.2%)	< 0.001
Total cholesterol (mmol/L)	338	4.1 [3.5;4.8]	4.2 [3.5;4.8]	4.1 [3.5;5.0]	4.0 [3.4;4.6]	0.60
HDL cholesterol (mmol/L)	337	1.1 [1.0;1.4]	1.1 [1.0;1.5]	1.2 [1.0;1.5]	1.1 [1.0;1.3]	0.124
Triglycerides (mmol/L)	338	1.2 [0.9;1.9]	1.2 [0.8;1.7]	1.2 [1.0;1.9]	1.5 [0.9;2.2]	0.01
LDL cholesterol (mmol/L)	336	2.2 [1.7;2.8]	2.3 [1.8;2.8]	2.2 [1.7;3.0]	2.1 [1.6;2.7]	0.41
Lipid-lowering treatment						
Statins	345	177 (51.3%)	53 (39.0%)	58 (56.9%)	66 (61.7%)	0.001
Fibrates	345	13 (3.8%)	3 (2.2%)	4 (3.9%)	6 (5.6%)	0.35
Ezetimibe	345	18 (5.2%)	5 (3.7%)	8 (7.8%)	5 (4.7%)	0.34
Current smoker	343	56 (16.3%)	29 (21.5%)	14 (13.9%)	13 (12.1%)	0.11
Aspirin consumption	341	60 (17.6%)	14 (10.4%)	17 (16.7%)	29 (27.6%)	0.002
Alcohol consumption	319					> 0.9
None		235 (73.7%)	92 (73.6%)	70 (72.2%)	73 (75.3%)	
< 3 drinks/day		69 (21.6%)	27 (21.6%)	22 (22.7%)	20 (20.6%)	
≥ 3 drinks/day		15 (4.7%)	6 (4.8%)	5 (5.2%)	4 (4.1%)	
*Computed tomography*						
Epicardial adipose tissue volume (cm^3^)	345	84.9 [61.8;114.3]	76.3 [50.3;100.6]	82.5 [64.4;107.3]	100.2 [77.3;134.6]	< 0.001
Coronary artery calcium score (AU)	345	0.7 [0.0;88.0]	0.0 [0.0;11.9]	0.8 [0.0;51.3]	46.4 [0.0;208.2]	< 0.001
Coronary artery calcium score > 100 AU	336	79 (23.5%)	19 (14.3%)	16 (16.3%)	44 (41.9%)	< 0.001
*pOpmetre®*						
Pulse wave velocity (m/sec)	345	8.0 [7.0;10.0]	6.2 [6.0;7.0]	8.3 [8.0;9.0]	11.0 [10.0;12.8]	< 0.001

*AU* Agatston unit, *CAD* Coronary artery disease, *PWV* Pulse wave velocity

*Hypertension and dyslipidemia were self-reported and/or inferred from prescriptions for antihypertensive and lipid-lowering agents, respectively

Data are given as the median [IQR], mean ± sd or n (%)

*p*-value: Fisher’s exact test or Chi-2 test for categorical variables and Kruskal–Wallis test for continuous variables

The mean (± SD) age of patients with T2D, T1D and other diabetes types was 59.0 ± 10.2, 45.2 ± 14.6 and 48.8 ± 10.6 years, respectively (*p* < 0.0001), while median [IQR] HbA1c was 8.3% [7.2–10.4], 8.2 [7.5–9.5] and 11.3 [9.8–12.4], respectively (p = 0.002). The median BMI differed significantly between diabetes types, with values of 29.0 kg/m^2^ [26.0; 33.0] in T2D, 26.0 [22.0; 30.0] in T1D, and 27.0 [24.0; 31.0] in other diabetes types (*p* < 0.001).

The median [IQR] EAT volume for the whole cohort was 84.9 cm^3^ [61.8–114.3] and median [IQR] PWV was 8.0 m/sec [7.0–10.0]. Subgroup analysis revealed that the median PWV was higher in patients with T2D (8.5 m/sec [7.0–10.0], Supplementary Table 1), compared to patients with T1D (6.8 m/sec [5.6–8.8], Supplementary Table 2) and with other types of diabetes (7.0 m/sec [6.0–8.0]). Moreover, 35.8%, 21.2% and 8.0% of patients with, respectively, T2D, T1D and other diabetes types, were in third PWV tertile (i.e., > 9 m/sec).

### Parameters associated with arterial stiffness

The higher the tertile the older the age, the longer the diabetes duration and the lower the HbA1c level. Ethnicity differed depending on PWV tertile (Table 1). Moreover, the higher the tertile, the higher the number of complications (retinopathy, albuminuria, neuropathy, macroangiopathy and high CAC score), cardiovascular risk factors (hypertension, dyslipidemia), diabetes-related treatments (metformin, sulfonylurea, glucagon-like peptide 1 receptor agonists), and antihypertensive treatments.

All these associations were globally similar in the 254 patients with T2D (Supplementary Table 1) and the 66 patients with T1D, particularly for age, hypertension and CAC score (Supplementary Table 2).

### Correlation between PWV and EAT across diabetes subtypes

**Fig. 1:**
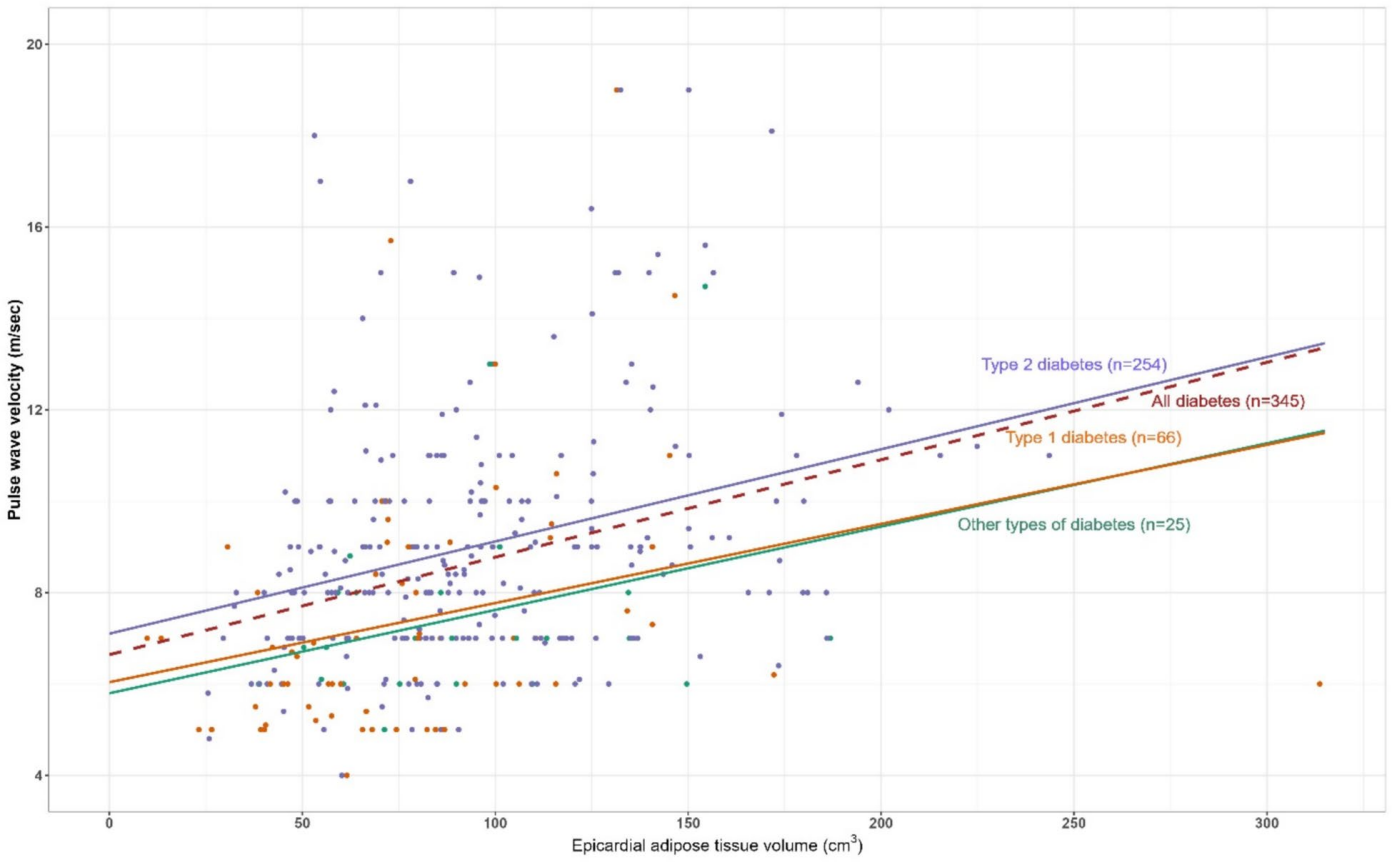
Scatter plot of epicardial adipose tissue volume and pulse wave velocity. The brown line shows the correlation for the whole population (Spearman correlation; r[95% CI] = 0.37[0.27–0.45]; *p* < 0.001), the purple line for patients with type 2 diabetes Spearman correlation; r[95% CI] = 0.31[0.19–0.42]; *p* < 0.001), the orange line for patients with type 1 diabetes (Spearman correlation; r[95% CI] = 0.42[0.20–0.61]; *p* < 0.001) and the green line for patients with other types of diabetes (Spearman correlation; r[95% CI] = 0.32[− 0.22–0.66]; p = 0.12)

As illustrated in Fig. 1, the Spearman correlation analysis revealed a significant positive association in the overall study population (*r* [95% CI] = 0.37 [0.27–0.45], *p* < 0.001). When stratified on diabetes type, a significant correlation was observed among patients with T2D (*r* [95% CI] = 0.31 [0.19–0.42], *p* < 0.001) and T1D (*r* [95% CI] = 0.42 [0.20–0.61], *p* < 0.001). In patients with other types of diabetes, the correlation was not statistically significant (*r* [95% CI] = 0.32 [− 0.22–0.66], p = 0.12).

**Fig. 2:**
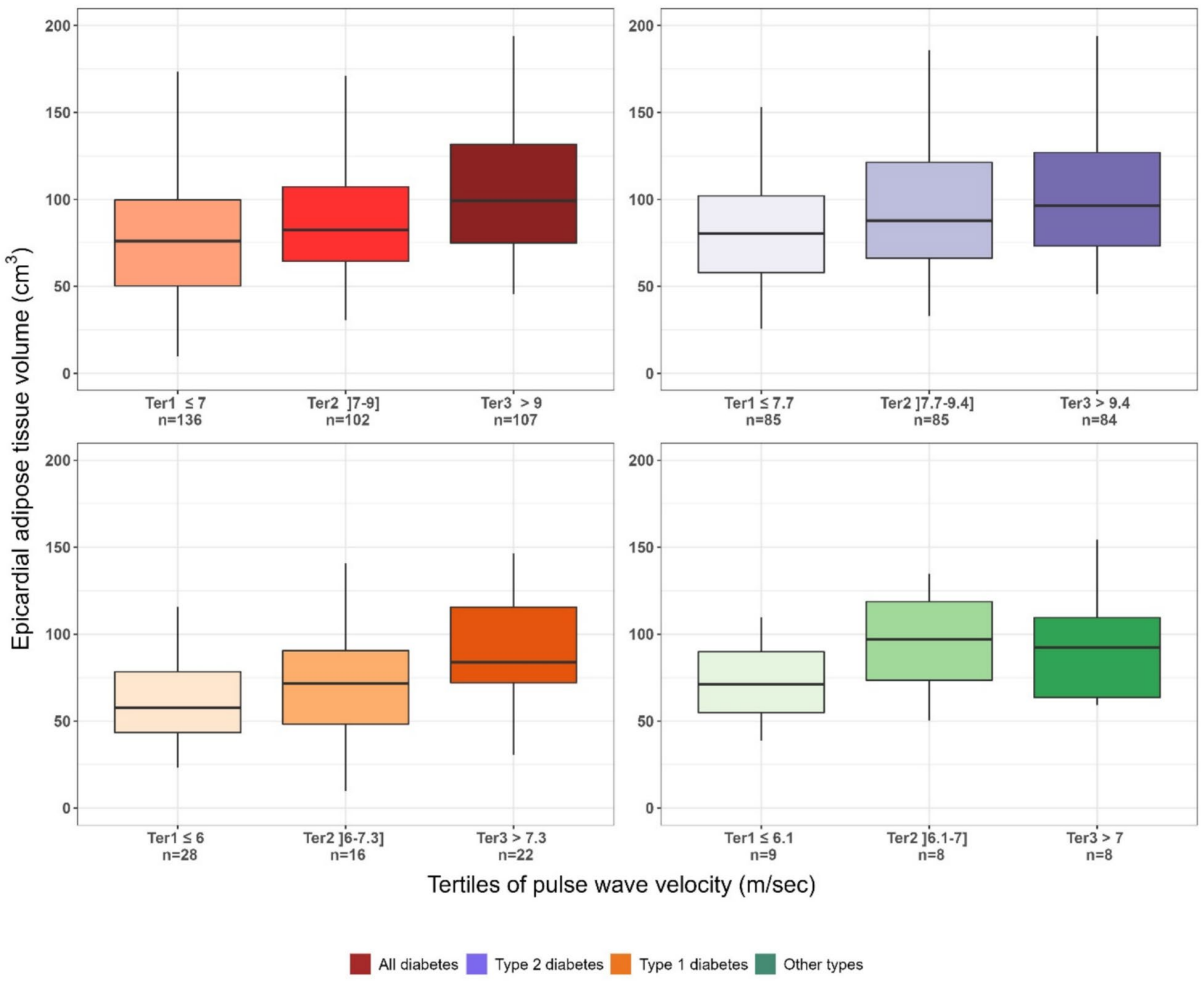
Boxplot of epicardial adipose tissue volume across pulse wave velocity tertiles. Boxplots display median, interquartile range, and extremes of epicardial adipose tissue volume as a function of pulse wave velocity tertiles; red for the whole population, purple for patients with type 2 diabetes, orange for patients with type 1 diabetes and green for patients with other types of diabetes

EAT volume increased significantly as a function of PWV tertile in the total cohort (*p* < 0.001; Fig. 2, Red; Table 1). It was also significantly higher in patients with T2D (Fig. 2, Purple; Supplementary Table 1) and T1D (Fig. 2, Orange; Supplementary Table 2). No significant increase was observed in patients with other types of diabetes (Fig. 2, Green).

### Factors associated with all participants in the third PWV tertile

The multivariable logistic models indicate that each 10 cm^3^ increase in EAT volume was associated with a 14% higher probability of belonging to the third tertile group, independently of age, mean central blood pressure, BMI and diabetes types (Model 1: OR = 1.14, 95% CI [1.06–1.21], *p* < 0.001). This positive association remained significant across all subsequent models, which incorporated additional covariates including ethnicity, lipid parameters, diabetes-related complications, hypertension status, and diabetes duration (Table 2). Notably, stratified analyses by obesity status revealed that the association was borderline significant among non-obese individuals (*p* = 0.06) and reached statistical significance in obese patients (*p* = 0.03).

**Table 2:** Association between epicardial adipose tissue volume and the third pulse wave velocity tertile (vs. combined first and second tertiles) in multivariable logistic analyses

Parameters	Adjustment for	Odds ratio (by 10 cm^3^ of EAT)	[95% confidence interval]	*p*
Model 1	EAT volume, age, mean central blood pressure, body mass index, type of diabetes	1.14	[1.06–1.21]	< 0.001
	Only persons without obesity: model 1 without body mass index	1.11	[1.00–1.23]	0.06
	Only persons with obesity: model 1 without body mass index	1.11	[1.01–1.21]	0.03
Model 2	Model 1 + ethnicity	1.13	[1.05–1.21]	0.001
Model 3	Model 1 + triglycerides, total and HDL cholesterol	1.12	[1.05–1.20]	0.001
Model 4	Model 1 + retinopathy, nephropathy and aspirin	1.16	[1.08–1.24]	< 0.0001
Model 5	EAT volume, age, hypertension, body mass index, type of diabetes	1.13	[1.06–1.21]	< 0.001
Model 6	EAT volume, diabetes duration, mean central blood pressure, body mass index, type of diabetes	1.19	[1.11–1.27]	< 0.0001

EAT: Epicardial adipose tissue.

## Discussion

Our cohort study results show that EAT volume was positively associated with arterial stiffness in persons with diabetes, and that this association remained significant after controlling for confounders.

Before the present work, only three other studies had suggested a correlation between the amount of EAT and arterial stiffness [19] or aortic compliance / distensibility [29, 30] in people with diabetes. Specifically, these studies included [19, 29] and 36 persons with diabetes [30], respectively. Therefore, the results could only be adjusted for a few potential confounders in the study samples, which included both people with T2D and controls [19, 29, 30]. Of note, one used transthoracic echocardiography to measure EAT thickness [29] while the others used cardiac magnetic resonance imaging to evaluate EAT surface area [19] or volume [30]. Our results, achieved using a CT scan to measure EAT volume and PWV (i.e., the gold standard) to measure arterial stiffness, and which were adjusted for many confounders, confirm these results in a larger series with well-phenotyped patients with T2D and T1D.

Several studies have also examined the relationship between EAT volume/thickness and arterial stiffness in different populations, including healthy people [20, 21], persons with hypertension [22, 23], individuals with ischemic heart disease [19, 24, 25], and patients with metabolic diseases such as non-alcoholic fatty liver disease [26] and inflammatory rheumatic diseases [27, 28]. While all these studies showed a positive correlation between EAT and arterial stiffness, many did not adjust for confounders [22, 25]. In three of them, EAT volume/thickness increased as the PWV class [20] or tertile [24] increased, in patients with a PWV above the median, and in those with a PWV ≥ 10 m/sec, respectively [28].

The median PWV of patients with T2D in our cohort was 8.5 m/sec. This is slightly lower than the 9.3 m/sec reported in a Rio de Janeiro study [37]. Additionally, median PWV was significantly lower in individuals with T1D (6.8 m/sec) compared to those with T2D. This reflects other studies which reported values of 8.1 m/sec [38] and 10.3 m/sec [39], respectively. The higher PWV observed in T2D may be attributable to a higher prevalence of associated cardiovascular risk factors, such as hypertension and dyslipidemia in this population. All these findings underscore the importance of monitoring arterial stiffness in persons with diabetes, particularly among those with T2D, to better manage and mitigate cardiovascular risks.

As previously reported in persons with diabetes [40–42], we found that older age, longer diabetes duration, a higher number of diabetes-related complications and of anti-hyperglycemic, antihypertensive and lipid-lowering treatments were all determinants of arterial stiffness. Furthermore, people with a higher PWV had a lower HbA1c. This finding differs from a Greek study which found no such association [39], and a Chinese study which found that a higher PWV was associated with higher HbA1c [29]. In our study, this may be due to the higher use of glucagon-like peptide-1 receptor agonists among individuals in the highest PWV tertiles.

Although our study was cross-sectional, and therefore could only show an association between EAT and arterial stiffness, some pathophysiological arguments may suggest a causal link between EAT and PWV. EAT is implicated in the pathogenesis of cardiovascular disease [43]. Moreover, in persons with diabetes, there is a relationship between EAT volume and CAC score [16], silent myocardial ischemia [17] and cardiac systolic dysfunction [44]. While the mechanisms involved in these three conditions are probably related to the paracrine effect of EAT [14], the association between EAT and arterial stiffness might suggest the presence of a systemic mechanism. EAT is a metabolically active endocrine organ and a source of pro-inflammatory adipokines and cytokines which, in addition to being diffused locally (paracrine effect), enter the bloodstream. The pathogenesis of the relationship between EAT and arterial stiffness has not been fully established, but the mechanisms involved may be related to vascular inflammation [19, 45] and endothelial dysfunction [27]. However, it is important to point out that a high EAT volume does not in itself mean that EAT is an inflammatory secretory phenotype. The vascular wall may also undergo atherogenic modifications through the activation of oxidative stress and lipolysis, as well as increased aggregation of activated macrophages with infiltration of macrophages and free fatty acids into the vascular endothelium [7]. Finally, EAT may contribute to vascular stiffness through other mechanisms, such as increased insulin resistance through reduced expression of glucose transporter 4, impaired endothelial-dependent vasodilation [24, 46], and the formation of advanced glycation end products in the vascular wall [41].

Conversely, an increase in EAT volume could coincide with an increase in other fat depots, such as pericardial adipose tissue [14]. In a series of 111 consecutive individuals undergoing CT scan, PWV was moderately associated with both EAT (r = 0.46, *p* < 0.001) and pericardial adipose tissue volume (r = 0.41, *p* < 0.001). After adjusting for conventional cardiovascular risk factors, the relative risk of the highest versus lowest PWV tertile was similar for significant EAT (3.03 [95% CI 1.22–7.51; p = 0.01]) and significant pericardial adipose tissue (2.34 [95% CI 1.10–4.90; p = 0.02]) [24]. As it lies outside the visceral pericardium, pericardial adipose tissue drains through lymphatics into the right atrium. Therefore, it is more likely than EAT to have systemic effects on the vasculature [14].

In addition to its cross-sectional nature, our study has other limitations. It was a monocentric study and the specific hospital setting might influence the generalizability of the findings. Moreover, we only included patients whose CAC score and PWV had been measured to estimate their cardiovascular risk. Of these, 10.8% had diagnosed macroangiopathy, a condition for which these two examinations are rarely performed. Therefore, our results may not be representative of all persons with diabetes. Although we could adjust for many potential confounders, some were unavailable, such including waist circumference and the characterization of pericardial [14] and perivascular adipose tissue [47]. On the other hand, our cohort was characterized by large ethnic diversity and patients with different types of diabetes. We used PWV to evaluate arterial stiffness, which is considered the gold standard [9–11] and CT scans to measure EAT volume, with automated quantification by dedicated software. This is more accurate than using ultrasound [16, 17, 32].

Our results suggest, in addition to earlier findings [4, 16, 17, 48], that measuring EAT volume might improve the assessment of cardiovascular risk. However, we do not know whether the prognostic value of EAT volume is cumulative to that of PWV measurement [4, 16]. Arterial stiffness is considered to be organ damage which in turn indicates a high cardiovascular risk [10, 12, 13, 18]. It is possible that a large EAT volume also reflects organ damage. However, there is currently no standard values for EAT volume. EAT volume can be modified by lifestyle (such as diet and/or exercise), bariatric surgery and pharmacological interventions [49, 50]. Accordingly, the existence of a causal link between a higher EAT volume and greater arterial stiffness could have therapeutic implications.

In conclusion, by showing an association between EAT volume and arterial stiffness in the population with diabetes independently of confounding factors, our study supports the hypothesis that EAT might affect arteries distant from the heart. Another hypothesis is that the increase in EAT is accompanied by an increase in other adipose tissues. Our finding suggests a potential underlying role of these tissues in inflammatory and metabolic mechanisms in exacerbating cardiovascular risk. Further research into these adipose depots as a biomarker for cardiovascular complications in people with diabetes is needed.

## Supplementary Information

Additional file 1

## Data Availability

Data for the present analysis can be provided from the first author on reasonable request.

## Abbreviations

95%CI: 95% Confidence interval
AU: Angstrom unit
BMI: Body mass index
CAC: Coronary artery calcium
CT: Computed tomography
EAT: Epicardial adipose tissue
eGFR: Estimated Glomerular filtration rate
ft-TT: Transit time between the toe and finger
GE: General electric
HU: Hounsfield unit
IQR: Inter quartile range
NCT: Clinical trial number
OR: Odds ratio
PACS: Picture archiving and communication systems
PWV: Pulse wave velocity
SD: Standard deviation
T1D: Type 1 diabetes
T2D: Type 2 diabetes
