# Supplementary material for: Epicardial adipose tissue volume and arterial stiffness in people living with diabetes: the METAB-CV-PWV study
Source: Cardiovasc Diabetol. 2025 Oct 15;24:400. doi: 10.1186/s12933-025-02933-z (PMC12522805; doi:10.1186/s12933-025-02933-z)
Supplement: Supplementary file 1 — Additional file 1 [file 12933_2025_2933_MOESM1_ESM.docx]

**Supplemental figure 1:** Study Flowchart


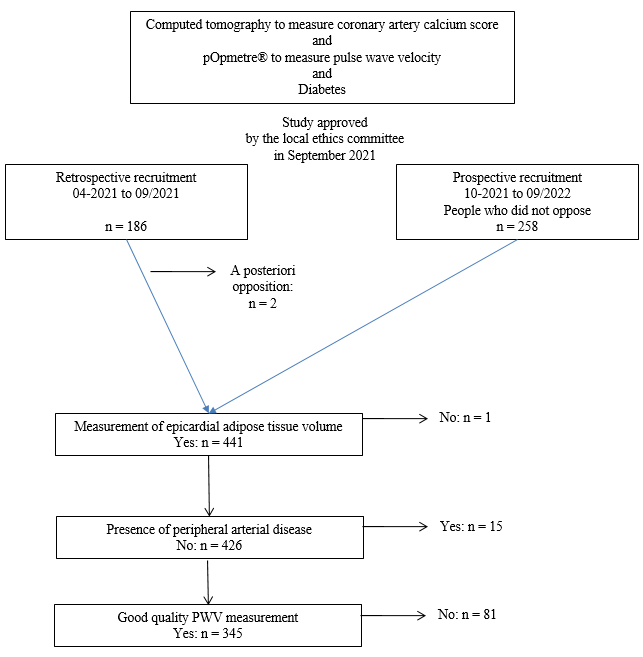


PWV: Pulse wave velocity

**Supplemental Figure 2:** characterization of epicardial adipose tissue volume

**
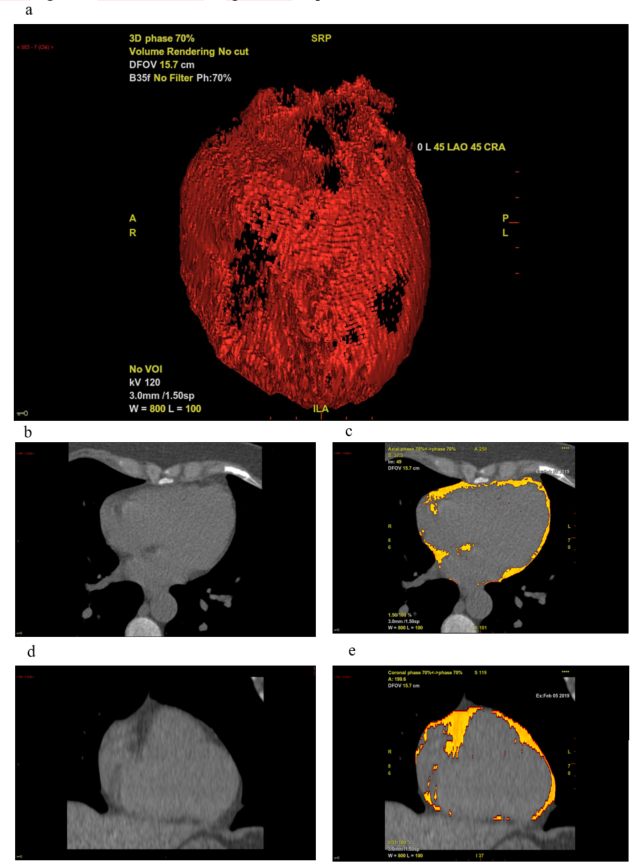
**a: three-dimension reconstitution of epicardial adipose tissue; example of axial image before (b) and after (c) epicardial adipose tissue identification; example of coronal image before (d) and after (e) epicardial adipose tissue identification

**Supplemental table 1**: Characteristics of patients with type 2 diabetes according to pulse wave velocity tertiles

|  | **Available data** | **Total** | **PWV**  **Tertile 1**  **≤ 7.7** | **PWV**  **Tertile 2**  **]7.7 – 9.4]** | **PWV**  **Tertile 3**  **> 9.4** | **p** |
| --- | --- | --- | --- | --- | --- | --- |
|  |  | n = 254 | n = 85 | n = 85 | n= 84 |  |
| **Clinical characteristics:** | | | | | | |
| Mean age ± SD (years) | 254 | 59.0 (10.2) | 54.7 (11.2) | 59.0 (9.3) | 63.4 (8.1) | <0.001 |
| Male sex | 254 | 135 (53.1%) | 43 (50.6%) | 48 (56.5%) | 44 (52.4%) | 0.73 |
| Obesity | 243 | 113 (46.5%) | 39 (49.4%) | 34 (42.0%) | 40 (48.2%) | 0.35 |
| Time since diagnosis (years) | 253 | 13.0 [7.0;20.0] | 10.0 [4.0;18.0] | 12.0 [8.0;19.0] | 17.0 [11.0;21.5] | <0.001 |
| HbA1c (%) | 212 | 8.3 [7.2;10.4] | 8.6 [7.2;12.1] | 8.4 [7.5;9.9] | 8.1 [7.1;9.8] | 0.18 |
| **Diabetes-related treatment:** | | | | | | |
| Metformin | 254 | 205 (80.7%) | 67 (78.8%) | 67 (78.8%) | 71 (84.5%) | 0.56 |
| Sulfonylurea | 254 | 113 (44.5%) | 32 (37.6%) | 41 (48.2%) | 40 (47.6%) | 0.30 |
| Alpha-glucosidase inhibitor | 254 | 8 (3.1%) | 3 (3.5%) | 3 (3.5%) | 2 (2.4%) | >0.9 |
| Di-peptidyl-peptidase 4 inhibitor | 254 | 72 (28.3%) | 24 (28.2%) | 28 (32.9%) | 20 (23.8%) | 0.42 |
| Sodium-glucose cotransporter-2 inhibitor | 254 | 18 (7.1%) | 5 (5.9%) | 6 (7.1%) | 7 (8.3%) | 0.83 |
| Glucagon-like peptide 1 receptor agonists | 254 | 82 (32.3%) | 22 (25.9%) | 28 (32.9%) | 32 (38.1%) | 0.23 |
| Insulin | 254 | 116 (45.7%) | 37 (43.5%) | 38 (44.7%) | 41 (48.8%) | 0.77 |
| **Diabetes-related complications:** | | | | | | |
| Retinopathy | 253 | 89 (35.2%) | 25 (29.4%) | 23 (27.1%) | 41 (49.4%) | 0.01 |
| Nephropathy | 253 | 69 (27.3%) | 21 (24.7%) | 21 (24.7%) | 27 (32.5%) | 0.79 |
| Neuropathy | 253 | 113 (44.7%) | 36 (42.4%) | 40 (47.6%) | 37 (44.0%) | 0.78 |
| Macroangiopathy | 251 | 29 (11.6%) | 4 (4.8%) | 9 (10.7%) | 16 (19.3%) | 0.01 |
| **Additional cardiovascular risk factors:** | | | | | | |
| Family history of premature CAD | 248 | 17 (6.9%) | 4 (5.0%) | 9 (10.6%) | 4 (4.8%) | 0.24 |
| Hypertension* | 253 | 153 (60.5%) | 43 (50.6%) | 50 (58.8%) | 60 (72.3%) | 0.02 |
| Central systolic blood pressure, mmHg | 227 | 137.0 [116.5;140.0] | 124.5 [109.0;140.0] | 137.0 [119.2;141.0] | 139.0 [124.5;140.5] | 0.005 |
| Central diastolic blood pressure, mmHg | 227 | 73.0 [72.0;78.0] | 73.5 [72.0;81.5] | 72.0 [72.0;76.5] | 73.0 [72.0;76.5] | 0.07 |
| Dyslipidemia* | 252 | 155 (61.5%) | 40 (48.2%) | 56 (65.9%) | 59 (70.2%) | 0.01 |
| Current smoker | 253 | 34 (13.4%) | 12 (14.1%) | 13 (15.5%) | 9 (10.7%) | 0.65 |
| **Computed tomography scan:** | | | | | | |
| Epicardial adipose tissue (cm^3^) | 254 | 87.4 [67.4;118.2] | 80.4 [57.9;102.0] | 87.8 [66.2;121.3] | 96.7 [75.7;132.9] | <0.001 |
| Coronary artery calcium score (AU) | 254 | 4.0 [0.0;134.3] | 0.0 [0.0;44.8] | 2.0 [0.0;63.5] | 93.5 [0.4;257.9] | <0.001 |
| **pOpmetre®:** | | | | | | |
| Pulse wave velocity (m/sec) | 254 | 8.5 [7.0;10.0] | 7.0 [6.0;7.0] | 8.5 [8.0;9.0] | 11.0 [10.0;12.7] | <0.001 |

AU: Agatston unit - CAD: coronary artery disease – PWV: pulse wave velocity

*Hypertension and dyslipidemia were self-reported and/or inferred from prescriptions for antihypertensive and lipid-lowering agents, respectively

Data are given as the median [IQR], mean ± sd or n (%).

p-value: Fisher's exact test or Chi-2 test for categorical variables and Kruskal-Wallis test for continuous variables

**Supplemental table 2**: Characteristics of patients with type 1 diabetes according to pulse wave velocity tertiles

|  | **Available data** | **Total** | **PWV**  **Tertile 1**  **≤ 6** | **PWV**  **Tertile 2**  **]6 -7.3]** | **PWV**  **Tertile 3**  **> 7.3** | **p** |  |
| --- | --- | --- | --- | --- | --- | --- | --- |
|  |  | n = 66 | n = 28 | n = 16 | n= 22 |  |  |
| **Clinical characteristics:** | | | | | | |  |
| Mean age ± SD (years) | 66 | 45.2 (14.6) | 41.9 (14.5) | 41.4 (12.5) | 52.0 (14.3) | 0.02 |  |
| Male sex | 66 | 44 (66.7%) | 15 (53.6%) | 12 (75.0%) | 17 (77.3%) | 0.15 |  |
| Obesity | 64 | 17 (26.6%) | 5 (17.9%) | 3 (21.4%) | 9 (40.9%) | 0.08 |  |
| Time since diagnosis (years) | 66 | 13.0 [7.0;22.0] | 16.0 [6.0;25.5] | 8.5 [6.2;13.0] | 18.0 [8.0;24.5] | 0.08 |  |
| HbA1c (%) | 53 | 8.2 [7.5;9.5] | 8.6 [7.5;9.6] | 8.1 [7.6;10.0] | 8.2 [7.5;9.4] | > 0.9 |  |
| **Diabetes-related complications:** | | | | | | | |
| Retinopathy | 66 | 28 (42.4%) | 12 (42.9%) | 5 (31.2%) | 11 (50.0%) | 0.24 |  |
| Nephropathy | 66 | 10 (15.2%) | 6 (21.4%) | 0 (0.0%) | 4 (18.2%) | 0.12 |  |
| Neuropathy | 66 | 12 (18.2%) | 3 (10.7%) | 4 (25.0%) | 5 (22.7%) | 0.40 |  |
| Macroangiopathy | 66 | 6 (9.1%) | 3 (10.7%) | 1 (6.2%) | 2 (9.1%) | > 0.9 |  |
| **Additional cardiovascular risk factors:** | | | | | | | |
| Family history of premature CAD | 66 | 2 (3.0%) | 1 (3.6%) | 1 (6.2%) | 0 (0.0%) | 0.71 |  |
| Hypertension* | 66 | 13 (19.7%) | 5 (17.9%) | 0 (0.0%) | 8 (36.4%) | 0.02 |  |
| Central systolic blood pressure, mmHg | 55 | 138.0 [133.0;140.0] | 137.0 [134.0;140.0] | 137.5 [113.8;139.2] | 139.0 [135.5;141.0] | 0.42 |  |
| Central diastolic blood pressure, mmHg | 55 | 73.0 [72.0;74.0] | 72.0 [72.0;74.0] | 73.0 [72.0;74.8] | 73.0 [72.0;74.0] | 0.58 |  |
| Mean central blood pressure, mmHg | 55 | 94.3 [92.5;95.7] | 94.0 [92.7;96.0] | 94.0 [90.2;95.0] | 95.0 [93.5;95.7] | 0.59 |  |
| Dyslipidemia* | 67 | 25 (37.3%) | 9 (32.1%) | 5 (29.4%) | 11 (50.0%) | 0.36 |  |
| Current smoker | 65 | 16 (24.6%) | 8 (29.6%) | 5 (31.2%) | 3 (13.6%) | 0.36 |  |
| **Computed tomography scan:** | | | | | | | |
| Epicardial adipose tissue (cm^3^) | 66 | 72.1 [52.0;100.2] | 58.9  [44.3;82.9] | 71.7  [48.3;90.6] | 83.9 [72.1;115.6] | 0.01 |  |
| Coronary artery calcium score (AU) | 66 | 0.0 [0.0;4.8] | 0.0 [0.0;0.2] | 0.0 [0.0;0.0] | 0.4 [0.0;37.3] | 0.07 |  |
| **pOpmetre®:** | | | | | | | |
| Pulse wave velocity (m/sec) | 66 | 6.8 [5.6;8.8] | 5.3 [5.0;6.0] | 7.0 [6.8;7.0] | 9.3 [9.0;10.5] | <0.001 |  |

AU: Agatston unit - CAD: coronary artery disease – PWV: pulse wave velocity

*Hypertension and dyslipidemia were self-reported and/or inferred from prescriptions for antihypertensive and lipid-lowering agents, respectively

Data are given as the median [IQR], mean ± sd or n (%).

p-value: Fisher's exact test or Chi-2 test for categorical variables and Kruskal-Wallis test for continuous variables
